# Single-molecule mitochondrial DNA sequencing shows no evidence of CpG methylation in human cells and tissues

**DOI:** 10.1093/nar/gkab1179

**Published:** 2021-11-29

**Authors:** Iacopo Bicci, Claudia Calabrese, Zoe J Golder, Aurora Gomez-Duran, Patrick F Chinnery

**Affiliations:** MRC-Mitochondrial Biology Unit, The Keith Peters Building, Cambridge CB2 0XY, UK; Department of Clinical Neurosciences, University of Cambridge, Cambridge Biomedical Campus, Hills Road, Cambridge CB2 0XY, UK; MRC-Mitochondrial Biology Unit, The Keith Peters Building, Cambridge CB2 0XY, UK; Department of Clinical Neurosciences, University of Cambridge, Cambridge Biomedical Campus, Hills Road, Cambridge CB2 0XY, UK; MRC-Mitochondrial Biology Unit, The Keith Peters Building, Cambridge CB2 0XY, UK; Department of Clinical Neurosciences, University of Cambridge, Cambridge Biomedical Campus, Hills Road, Cambridge CB2 0XY, UK; MRC-Mitochondrial Biology Unit, The Keith Peters Building, Cambridge CB2 0XY, UK; Department of Clinical Neurosciences, University of Cambridge, Cambridge Biomedical Campus, Hills Road, Cambridge CB2 0XY, UK; Centro de Investigaciones Biológicas Margarita Salas. Spanish National Research Council, Madrid, Spain; MRC-Mitochondrial Biology Unit, The Keith Peters Building, Cambridge CB2 0XY, UK; Department of Clinical Neurosciences, University of Cambridge, Cambridge Biomedical Campus, Hills Road, Cambridge CB2 0XY, UK

## Abstract

Methylation on CpG residues is one of the most important epigenetic modifications of nuclear DNA, regulating gene expression. Methylation of mitochondrial DNA (mtDNA) has been studied using whole genome bisulfite sequencing (WGBS), but recent evidence has uncovered technical issues which introduce a potential bias during methylation quantification. Here, we validate the technical concerns of WGBS, and develop and assess the accuracy of a new protocol for mtDNA nucleotide variant-specific methylation using single-molecule Oxford Nanopore Sequencing (ONS). Our approach circumvents confounders by enriching for full-length molecules over nuclear DNA. Variant calling analysis against showed that 99.5% of homoplasmic mtDNA variants can be reliably identified providing there is adequate sequencing depth. We show that some of the mtDNA methylation signal detected by ONS is due to sequence-specific false positives introduced by the technique. The residual signal was observed across several human primary and cancer cell lines and multiple human tissues, but was always below the error threshold modelled using negative controls. We conclude that there is no evidence for CpG methylation in human mtDNA, thus resolving previous controversies. Additionally, we developed a reliable protocol to study epigenetic modifications of mtDNA at single-molecule and single-base resolution, with potential applications beyond CpG methylation.

## INTRODUCTION

Cytosine methylation is an epigenetic modification of nuclear DNA (nDNA) that can regulate gene expression during development (1) and throughout life (2), but the presence of CpG methylation on the mitochondrial genome (mtDNA) is a matter of debate (3–5). This is an important issue to resolve given the pivotal role of mtDNA in cellular metabolism (6).

Whole genome bisulfite sequencing (WGBS) is the gold standard technique for detecting methylation across the nuclear genome (7–9), where sequencing before and after the chemical conversion of unmethylated cytosine to uracil allows the degree of methylation to be measured at single-base resolution. WGBS studies have reported methylation patterns across the mtDNA molecule in different biological contexts (10). However, recent studies suggest that these are influenced by technical artefacts (3–5,11). MtDNA has a purine-rich ‘Heavy’(H-) and a pyrimidine-rich ‘Light’(L-) strand (12), leading to a disproportionate fragmentation of the cytosine-rich L-strand by bisulfite treatment (13). Moreover, the presence of multiple mtDNA genotypes within mitochondria of the same cell (heteroplasmy (14)), and nuclear sequences originated from the mtDNA (NuMTs (15,16)) are potential confounders for mtDNA methylation detection.

To overcome these limitations, we set out to quantify CpG methylation of native mtDNA using long-read based Oxford Nanopore Sequencing (ONS) technology (17). We developed a new protocol enabling the assessment of methylation at nucleotide-level resolution (18), to study multiple human cell lines and in human tissues. We show that the population nucleotide sequence variants introduce artifacts giving the impression of mtDNA methylation which can be removed using an individual-specific mtDNA reference sequence. We also show that residual apparent low levels of mtDNA methylation fall below the detection threshold for ONS, providing independent evidence that significant levels of mtDNA methylation is unlikely to occur *in vivo*.

## MATERIALS AND METHODS

### Cell culture and DNA extraction from human cell lines

Cell lines used in this study are listed in Table 1. Cells were maintained in fibroblast medium [DMEM high glucose (Gibco) with 10% foetal bovine serum (Gibco) and no antibiotics] at 37°C in a humidified 5% CO_2_ atmosphere. Cells were grown until ∼80% confluence. When ready, cells were washed with PBS (Gibco), then incubated with 0.05% trypsin (Gibco) for 5 min at 37°C. Cells were collected by centrifugation (1500 rcf for 5 min) and pellets were washed once with PBS, before being snap frozen in liquid nitrogen and kept at −20°C until further use. All DNA from cell lines was extracted from snap-frozen pellets using the QIAmp DNA mini kit (QIAGEN) following the manufacturer's instructions. DNA was quantified using the Qubit dsDNA kit (Invitrogen) following the manufacturer's instructions.

**Table 1.** List of cells and tissues used in this study

**Cell line studied**					
**Cell line description**	**Code**	**Used for**		**ONS protocol**	
Human cybrid cell line - H haplogroup	613H	ONS library preparation, variant calling, methylation analysis		Fragmentation, BamHI-based	
Human cybrid cell line - J haplogroup	128J	ONS library preparation, variant calling, methylation analysis		Fragmentation, BamHI-based	
Human cybrid cell line - J2 haplogroup	135J2	ONS library preparation, variant calling, methylation analysis		Fragmentation, BamHI-based	
Human primary fibroblast cell line - Control	Control 1	Variant calling, methylation analysis		BamHI-based	
Human primary fibroblast cell line - Control	Control 2	Variant calling, methylation analysis		BamHI-based	
Human primary fibroblast cell line - MELAS mutation	m.3243A>G (1)	Variant calling, methylation analysis		BamHI-based	
Human primary fibroblast cell line - MELAS mutation	m.3243A>G (2)	Variant calling, methylation analysis		BamHI-based	
Human primary fibroblast cell line - MERRF cell line	m.8344A>G	Variant calling, methylation analysis		BamHI-based	
**Human tissues studied**					
**Tissue type**	**ID code**	**Source**	**Gender/age**	**Used for**	**ONS protocol**
Human Liver	TB15-0139	Addenbrooke's Tissue Bank	Male/36 years	Methylation analysis	BamHI-based
Kidney	TB12-1905	Addenbrooke's Tissue Bank	Male/60 years	Methylation analysis	BamHI-based
Human Kidney	TB15-153	Addenbrooke's Tissue Bank	Male/75 years	Methylation analysis	BamHI-based
Heart	TB12-2860	Addenbrooke's Tissue Bank	Male/28 years	Methylation analysis	BamHI-based
Skeletal Muscle	TB15-2606	Addenbrooke's Tissue Bank	Male/56 years	Methylation analysis	BamHI-based
Skeletal Muscle	TB13-1505	Addenbrooke's Tissue Bank	Male/40 years	Methylation analysis	BamHI-based
Skeletal Muscle	TB05-0578	Addenbrooke's Tissue Bank	Male/82 years	Methylation analysis	BamHI-based

### DNA extraction from human tissues

Tissues used in this study are listed in Table 1. Tissues were obtained from 7 different healthy individuals. All DNA from human tissues was extracted using QIAmp Fast DNA Tissue Kit (QIAGEN), following the manufacturer's instructions. DNA was quantified using the Qubit dsDNA kit (Invitrogen) following the manufacturer's instructions.

### Long-range polymerase reactions (LR-PCR)

LR-PCR amplification reaction was performed using PrimeSTAR GXL DNA Polymerase kit (Takara) according to manufacturer's instructions. The primers used are detailed in Supplementary Table S1. Product length encompasses most part of the mtDNA sequence. Amplification reactions were performed using the following cycling conditions: 94°C for 1 min, followed by 30 cycles of 98°C for 10 s, 55°C for 15 s and 68°C for 10 min.

### Generation of negative and positive controls

Untreated LR-PCR amplicons were used as negative controls for methylation. To generate positive controls, the same amplicons were treated *in vitro* with the recombinant CpG methyltransferase M.SssI (NEB). Briefly, 1 μg of amplicon DNA per 50μl reaction was treated for 4 h at 37°C with 50 units of M.SssI in the presence of 1× NEB buffer #2 and 160μM of S-adenosylmethionine (SAM). To test the efficiency of the M.SssI reaction, 10 units of methylation-sensitive restriction enzyme BstUI were added at the end of the incubation. This was followed by a further incubation at 60°C for 1 h. Protection of the M.SssI-treated amplicons from BstUI digestion was assessed using the Genomic DNA ScreenTape System (Agilent) on an Agilent 2200 TapeStation platform following manufacturer's instructions (data not shown). Supplementary Figure S1. To generate positive controls with intermediate methylation levels, we mixed negative and positive controls according to Supplementary Table S2.

### Mitochondrial DNA enrichment for single-molecule sequencing

1 μg of genomic DNA (nuclear + mitochondrial DNA) per 50 μl reactions was digested with 40 units of the recombinant restriction enzyme BamHI-HF (NEB) for 1 h at 37°C in the presence of CutSmart buffer (NEB). To achieve combined DNA purification and selection of high molecular weight fragments, DNA was purified using Monarch^®^ PCR & DNA Cleanup Kit (NEB), using the following recommended protocol modification: 20 μl of elution buffer was heated to 50°C before the last elution step.

### Quantification of mtDNA levels using ddPCR

ddPCR was used to quantify relative mtDNA enrichment following BamHI-HF (NEB) treatment of control DNA. To quantify relative mtDNA copy number, a mitochondrial and nuclear target (the genes *MT-ND1* and *RNASE P*, respectively) were amplified and fluorescent signal was generated using the primers and probes detailed in the Supplementary Table S1. ddPCR protocol was performed following manufacturer's instructions. Briefly, PCR reaction master mix was prepared in 1x (final concentration) ddPCR Supermix for Probes (no dUTP, BioRad), by adding 300nM of each primer and 200 nM of each probe in 19 μl final volume. 1 ng of sample DNA was then added to the mastermix. Droplets were generated using an Automated Droplet Generation instrument (BioRad) and were then subjected to PCR amplification, performed using the following cycling conditions: 95°C for 10 min, followed by 39 cycles of 94°C for 30 s and 58°C for 1 min, followed by a final stabilization step at 98°C for 10 min. Droplets were then loaded into a QX200 droplet reader (BioRad) and analysed using an absolute quantification protocol (ABS) to measure the absolute copy number of each probe. Droplet analysis was performed using the QuantaSoft analysis software (BioRad).

### ONS library preparation and sequencing on the MinION instrument

Approximately 1 μg of native genomic DNA or purified LR-PCR amplicons were prepared for ONS sequencing on R9.4.1 flow cells using the Ligation Sequencing Kit SQK-LSK109 (Nanoporetech), in combination with the Native Barcoding Expansion Kit EXP-NBD114 (Nanoporetech). Genomic DNA was fragmented either through BamHI digestion (Materials and Methods) or sheared to 10 kb using g-tubes (Covaris), following manufacturers’ instructions. Simultaneous DNA repairing, end-repairing and dA-tailing was achieved using the NEBNext FFPE Repair Mix (NEB) and the Ultra II end-repair module (NEB). Barcodes were ligated to individual samples using Blunt/TA Ligase Master Mix (NEB). Samples were then combined and AMII adapters containing the motor proteins needed for sequencing were ligated using NEBNext^®^ Quick Ligation Module (NEB). AMPure XP beads (Beckman Coulter) at a concentration of 1x, 1x and 0.5x, respectively, were used to purify DNA between the library preparation steps. Final libraries were loaded onto R9.4.1 flow cells and samples were sequenced using a single MinION Mk 1B. To keep the sequencing throughput consistent, six biological samples were always pooled together and sequenced for 24 h. LR-PCR amplicons were pooled together and sequenced for 6 h.

### Illumina Miseq library preparation and sequencing

MiSeq libraries were prepared from genomic DNA by amplification of the mitochondrial DNA in two overlapping fragments, using the primers shown in Supplementary Table S1. Amplicons were individually purified, quantified, and then were pooled in equal amounts from each sample. Libraries were prepared using NEBNext Ultra library prep reagents (NEB) according to manufacturer's instructions and sequenced using a 2 × 250-cycle MiSeq Reagent kit v3.0 (Illumina, CA).

### WGBS data analysis

Raw WGBS experiments part of the Roadmap Epigenome Project (19) were downloaded from the GEO Database. Downloaded files from single-ended WGBS sequencing experiments were converted from SRA format to fastq files using fastq-dump (Key Resources Table) with the following options: –readids –skip-technical -W –read-filter pass –gzip. Read quality of the converted fastq files was assessed with FastQC v0.11.5 (20). All of the reports generated from FastQC were manually checked to determine whether a trimming of low-quality reads and/or adapters was needed. Where trimming was deemed necessary, TrimGalore! v0.4.5 (https://www.bioinformatics.babraham.ac.uk/projects/trim_galore/) was used. The software automatically trims adapter sequences from the reads (if present) and retains those with an average Phred quality score ≤20 (before and/or after trimming). Reads shorter than 45 bp after trimming were discarded using –length option. Upon quality check and trimming, both alignment of the WGBS fastq files to the reference human genome sequence (GRCh38) and extraction of the methylation information were carried out with bowtie2 v2.3.2 (21) and Bismark v0.19.0 (22), respectively. Coverage was calculated from BAM files using samtools depth. This was defined as the percentage of mtDNA genome in each strand covered by at least 5 reads. Methylation extraction was carried out using the bismark_methylation_extractor package with the following options: –comprehensive –merge_non_CpG –gzip –bedGraph –CX_context, but only CpG residues were considered for further analyses.

### ONS data analysis

Base-calling of fast5 files containing raw electric current information was performed by the guppy_basecaller package of Guppy v3.2.2+9fe0a78 (Nanoporetech). Base-called, barcoded reads were de-multiplexed into individual samples using the guppy_barcoder package of Guppy v3.2.2+9fe0a78 (Nanoporetech). In order to simultaneously enrich for linear full-length mitochondrial sequences, exclude ligation artifacts and minimise the presence of NuMTs, we applied a stringent filter on read sequence length (min: 4000 bp, max: 17 000 bp) and quality (Phred quality score ≥ 9) using NanoFilt v2.2.0 (23) on the barcoded fastq files.

Minimap2 v2.10-r761 (24) with the -x map-ont option was used to perform the alignment of Nanopore reads onto the GRCh38 reference (which includes the mitochondrial rCRS reference sequence, NC_012920.1), and the option -secondary = no was used to exclude secondary alignments in the BAM output. Because minimap2 does not recognise circular reference sequences, reads spanning the D-loop are reported as supplementary alignments in the output BAM files. For this reason, we included in the final set of aligned reads also supplementary alignments aligning onto the mtDNA reference and spanning the D-loop, but only if they aligned in the same orientation on the same strand (H or L strand). Any other kind of supplementary alignment was excluded. Similarly, to avoid the same issue with reads spanning the BamHI cut site in the *ND6* gene (base 14 258–14 259 of the mtDNA reference sequence), we created an alternative GRCh38 reference sequence with a modified mitochondrial reference starting at base 14 259 instead of base 1. All of the experiments where the samples were fragmented using BamHI were aligned to this alternative sequence (gene annotations were adapted accordingly). Quality control plots and sequencing statistics were automatically generated using NanoPlot v1.13.0 (23).

### ROC curve generation

We calculated a ROC curve to assess the accuracy of our methylation calling, using a procedure previously adopted in Simpson et al (25). Briefly, we randomly chose 50,000 mtDNA CpG sites from positive and negative controls and classified each CpG call as true positive (TP) or false positive (FP), depending on which of the two controls each site came from and on whether methylation fell above or below a log-likelihood methylation threshold. We repeated the TP and FP calculation by varying log-likelihood threshold values within a range of –20 to 20 (to build the ROC curve) and 0 to 10 (to calculate accuracy, intended as the proportion of true calls, either TP or true negatives (TN)), with a step of 0.25, as explained in Simpson *et al.* (25).

### Dataset simulation and background noise modelling

To elucidate the relationship between the methylation levels and the read depth in ONS data, we generated *in silico* multiple datasets of simulated sequencing experiments, subsampling the negative control BAM file. We used samtools -s (read fraction) -b BAM > simulated.sam. We selected 30 different read fractions matching the read depths achieved with both the fragmentation and BamHI-based sequencing experiments on native DNA. Once the simulated SAM files were generated, we proceeded with the methylation calling using Nanopolish, following the same workflow used for cell lines, primary fibroblasts and tissues. Methylation levels calculated on the simulated data were therefore considered background noise introduced by either the ONS technique or the methylation calling procedure. We chose a function describing an exponential decay (1) to model the background noise, given the inverse relationship we observed in simulated data (high methylation levels corresponding to low read depth and vice versa).

(1) $$\begin{equation*}Y = m*{e^{\left( { - t*x} \right)}} + b\end{equation*}$$

The goodness of fit test showed that the exponential function in (1) well explained the variation of the simulated data (*R*^2^ = 0.94), therefore we set out to use the estimated parameters (*m*, *t* and *b*) and the equation in (1) to calculate the background noise present in all downstream ONS sequencing experiments. The background noise model fitting was performed using the optimize.curve_fit function of the Scipy Python module. All analyses have been performed in Python 3.0 and code is available at https://github.com/ib361/scripts_paper.

### Mitochondrial variant calling of ONS samples

Because Nanopore technology allows a simultaneous read of epigenetic modifications while sequencing the target DNA, we performed a mitochondrial variant calling on the fastq files filtered with NanoFilt v2.2.0 (23). For this we used a modified version of the MToolBox pipeline (26), adapted to long-reads sequencing analysis (https://github.com/mitoNGS/MToolBox/tree/MToolBox_Nanopore). Briefly, the main changes integrated into the MToolBox workflow are (i) the use of the Minimap2 aligner software (24) for long-reads mapping and (ii) additional parsing of SAM files to include reads uniquely mapped on the mtDNA reference and reads with supplementary alignments but only showing mtDNA mapping locations. These reads can be the results of the process of linearization of the circular molecule of mtDNA due to random fragmentation or to BamHI enzymatic cut. Reads with secondary or supplementary alignments on the nuclear genome were excluded and classified as possible NuMTs. For read mapping we used the GRCh38 human genome assembly (which includes rCRS as mitochondrial reference sequence). For variant calling, we set the quality score (QS) threshold to retain variants to 10 (changing the -q option of the assemblyMTgenome.py script). Variants with a read depth per position ≥30 and variant allele fraction ≥ 10% were retained. Only single nucleotide variants (SNVs) were considered for comparison with Illumina Miseq sequencing. Haplogroup predictions were performed using both MToolBox and Haplogrep 2 v.2.1.1 (27). Haplogrep2 predictions were based on homoplasmic variants only (with variant allele fraction ≥ 0.9).

### Mitochondrial variant calling of Illumina Miseq samples

Fastq files generated with Illumina Miseq were checked for quality using FastQC v0.11.5 (20). Illumina adapters and read ends showing poor *per*-base quality were trimmed using TrimGalore! v0.4.5 (https://www.bioinformatics.babraham.ac.uk/projects/trim_galore/), setting a minimum per-base QS = 20 and minimum read length after trimming = 35 bp. Mitochondrial variant calling was then performed with the standard MToolBox pipeline (26), which mapped reads to the human reference genome (GRCh38) with the two-mapping step protocol, to exclude possible NUMT. Single nucleotide variants with ≥5 reads of support (and at least 1 read of support on each strand) and minimum QS per base ≥25 were retained. Haplogroup predictions were performed using both MToolBox and Haplogrep 2 v.2.1.1 (27). Haplogrep2 predictions were based on homoplasmic variants only (with variant allele fraction ≥ 0.9).

### CpG methylation detection in ONS samples

Detection of methylation in CpG context was carried out using Nanopolish v0.11.0 call-methylation package (25). Nanopolish utilises a trained Hidden Markov Model to detect modified bases by comparing raw electric signals of modified/unmodified cytosines with expected signal from a reference sequence. The methylation calling output is a log-likelihood ratio where a positive value indicates evidence supporting methylation. Nanopolish utilises fast5 files containing raw electric signal information, basecalled fastq files and BAM alignment files to generate an index file used by the algorithm to determine methylation Log-likelihood ratios. Minimap2 alignments to reference sequences were performed with the same parameters described in the Nanopore Data Analysis section. Log-likelihood ratios were then converted to a binary methylated/unmethylated call for each read, then percentage of methylation was obtained by calculating the fraction of methylated reads, using the calculate_methylation_frequency.py script available in the package. The default calling threshold of ≥2.5 LLR was modified to a more stringent ≥5 LLR to increase the accuracy of the call. Since Nanopolish groups neighbouring CpG sites and calls them jointly, CpG sites in the same group were separated and assigned the same methylation frequency using the -s option.

### CpG methylation analysis in ONS samples

We applied a series of stringent quality filters to remove possible artefacts of the CpG methylation calling and errors introduced by the Nanopolish algorithm. Then, we removed CpGs calls with a methylation frequency greater than two standard deviations from the mean in negative controls (false positives (Supplementary Table S3) and calls neighbouring any heteroplasmic nucleotide variant (heteroplasmy < 0.9) in a ±5 nucleotides window. This last approach was deemed necessary after noticing that Nanopolish introduced a false methylation call every time a homoplasmic haplogroup-defining variant position fell within ±5 nucleotides from a CpG. As 11 nucleotides is the kmer size that Nanopolish considers to calculate CpG LLR, we hypothesized that the introduction of a nucleotide variant within ±5 nucleotides from the CpG altered the Nanopolish methylation determination, leading to an incorrect methylation call. To demonstrate this, we used MToolBox (26) to generate a consensus sequence from the Illumina data, carrying the major alleles at each position, and used this new sequence to perform the methylation calling again on ONS samples. As expected, this time no methylation was identified in the CpGs close to the haplogroup-defining variants. Differential methylation analysis was performed using the R package DSS (28), following the protocol detailed in Gigante et al (18). Differentially methylated mtDNA positions and regions (defined by overlapping tiles of 50nt) were deemed significant if False Discovery Rate was below 1%. For the comparison of the human cell lines and the primary fibroblasts we used as baseline the 613H cell line and the control fibroblasts, respectively.

### Statistical tests

Each data distribution was checked for normality by using the Shapiro-Wilk test. For pairwise comparisons, we chose to use the parametric Student's *t*-test or Anova one-way test when values were normally distributed. When not stated, distributions were non-normal and a Wilcoxon two-tailed test was used instead. Spearman's rank test has been used to calculate correlation between variables.

## RESULTS

### CpG methylation analysis of mtDNA with WGBS

**Figure 1.**
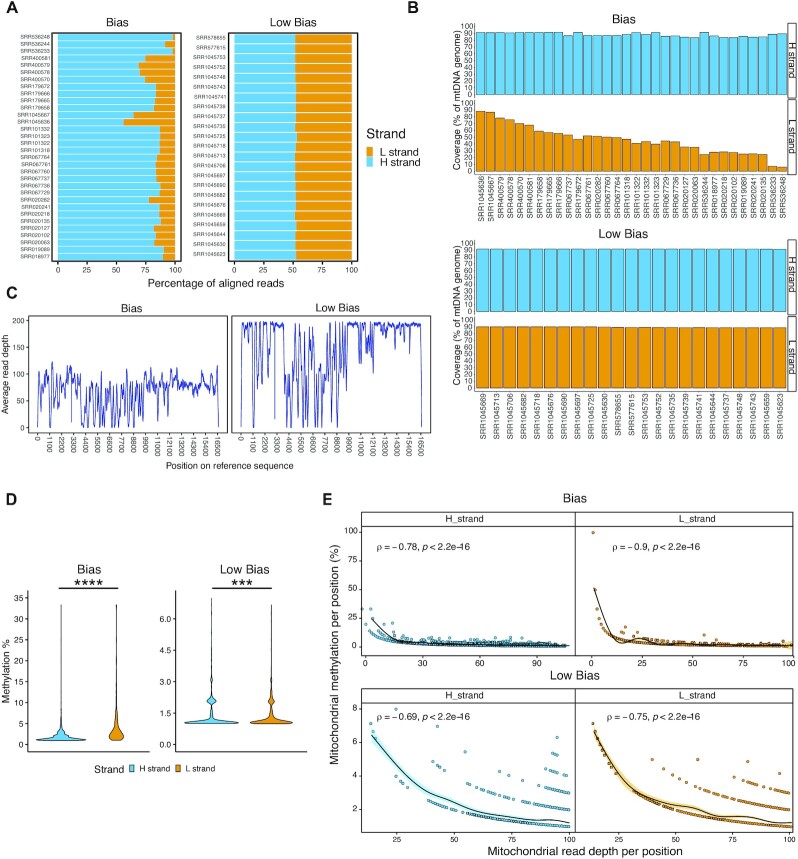
Quantification of WGBS alignment and coverage bias. (**A**) Percentage of reads aligned to the mtDNA reference per sample, identifying samples with a marked (bias, *N* = 32/55) or low (low bias, *N* = 23/55) per-strand-bias. (**B**) Percentage of mtDNA covered by at least 5 reads on the two mtDNA strands (H and L) in (top) bias and (bottom) low bias sample groups. (**C**) Distribution of the average read depth per mtDNA position in the two per-strand-bias groups. (**D**) Quantification of the average CpG methylation per strand (H and L), divided by per-strand-bias group. The lower and upper hinges of the violinplot correspond to the first and third quartile of the distribution, with median in the centre. Stars indicate significance (***: two-sided *P* ≤ 0.001; two-sided ****P ≤ 0.0001, Wilcoxon test). (**E**) Correlations between average read depth and average methylation percentage for every cytosine in CpG context, in the two per-strand-bias groups and mtDNA strands (H and L). Spearman's rank test correlation coefficient and two-sided P-values are shown. For all the plots in (D, E), average methylation is intended as the mean methylation value across all the WGBS samples analysed (*N* = 55).

We sought independent evidence that WGBS has limitations for mtDNA by analysing data from 67 human cell lines and tissues from the NIH Human Epigenome Roadmap Project (19). Fifty-five passed quality control (Materials and Methods) and were aligned to the human genome build GRCh38 (Supplementary Table S4). Analysis of the mtDNA-aligned reads revealed a pronounced *per*-strand mapping and coverage bias. By looking at the percentage of reads mapped *per*-mtDNA-strand, we arbitrarily divided the samples in two groups, depending on the proportion of reads mapping on the H strand (‘Biased’, where ≥55% of the reads aligned to on strand, and ‘Low Bias’ where 50–55% of the reads aligned to one strand). The Biased (BG) group included 58.2% (*N* = 32/55) samples with the majority of reads mapped to the mitochondrial H-strand (*P* ≤ 0.0001, Figure 1A, Supplementary Figure S2A) and a more pronounced *per*-strand coverage bias on the L-strand (L-strand coverage_BG_ = 6.2–88.3%; H-strand coverage_BG_ = 83.5–91.7%, Figure 1B top panel). The remaining data (*N* = 23/55, ‘Low Bias’ group, LBG), showed less mapping bias on the H-strand (between 50-55% reads; *P* ≤ 0.0001, Figure 1A, Supplementary Figure S2A) but no coverage bias (Figure 1B, bottom panel). We observed differences (*P* ≤ 0.0001) in the average read depth per position calculated in the two groups: 66.32 ± 28.84× BG versus 148.77 ± 55.45× LBG (group mean ± sd; Figure 1C). We found higher apparent methylation levels in the L-strand compared to the H in all samples analysed (L-strand_BG_ = 4.97% ± 8.79 versus H-strand_BG_ = 2.01% ± 1.92 mean methylation ± sd; L-strand_LBG_ = 1.43% ± 0.77 versus H-strand_LBG_ = 1.39% ± 0.7 mean methylation ± sd; *P* ≤ 0.001; Figure 1D, Supplementary Figure S2B). This is explained by a significant inverse correlation with the read depth per position, leading to the appearance of higher methylation levels where the read depth is low (Spearman's rank test *P* < 2.2e−16; average rho coefficient = −0.78, Figure 1E). This holds true also for the Low Bias group (with less alignment bias), where local fluctuations in the read depth alter CpG methylation levels (Supplementary Figure S2C, D). This is consistent with previous observations indicating a bisulfite-related selective loss of the cytosine-rich L-strand (13).

### Design and assessment of an ONS-based protocol for mtDNA enrichment and analysis

To overcome the problems intrinsic to the WGBS methylation determination, we set out to quantify mtDNA CpG methylation using ONS on native human gDNA. First, we developed a custom-made library preparation protocol (Supplementary Figure S3A) based on the simultaneous linearisation and enrichment of the native full-length mtDNA molecule (Supplementary Figure S3B) through BamHI restriction enzyme digestion (which usually cuts the mtDNA once). We tested the efficiency of our modified protocol over the standard ONS library preparation based on random fragmentation, by performing ONS on gDNA from 3 trans-mitochondrial osteosarcoma cybrid cell lines with known mtDNAs belonging to different mtDNA human haplogroups (29) and with an identical nuclear background (30) (N = 5 biological replicates of 3 independent cell lines with the mitochondrial haplogroup H1, J1c and J2, respectively; ‘613H’, ‘128J’, ‘135J2’; Table 1, Supplementary Table S5). Each gDNA was processed in parallel with both protocols. We further performed strict filtering on read lengths (selecting between 4000 and 17 000 bp) and per read quality (Phred ≥ 9) before the alignment (Supplementary Figure S3C-D and S4A-B), followed by supplementary alignment removal. This filtering excludes the possibility of studying mitochondrial 7s DNA (31), as its average length (∼650 bp) falls below our minimum read length threshold. While not altering quality parameters (percentage of identity and average base quality *per*-read, Supplementary Figure S4C, D), our filtering enriched for full length mtDNA sequences in all BamHI-treated samples. A higher percentage of reads aligned on mtDNA in the BamHI protocols compared to the fragmentation protocol, further confirming that the BamHI treatment enriched for mtDNA (Student's *t*-test *P* ≤ 0.05, Supplementary Figure S5).

**Figure 2.**
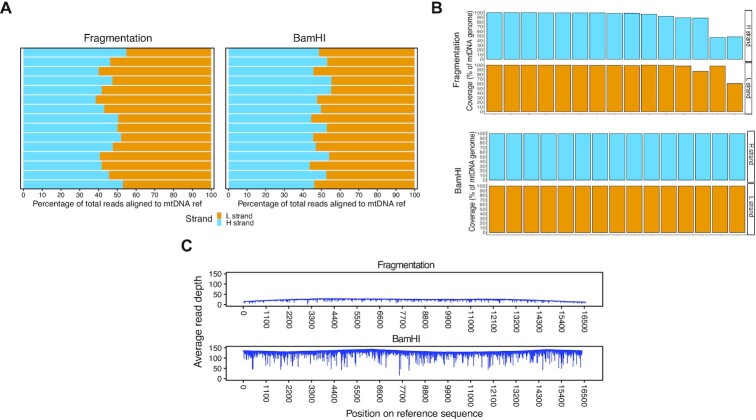
BamHI-based protocol improves mtDNA reads alignment over the standard ONS library preparation. (**A**) Percentage of reads aligned to the mtDNA reference per strand per biological replicate (*N* = 15 samples per protocol), in samples processed with (left) fragmentation protocol and (right) BamHI-based protocol. (**B**) Percentage of mtDNA covered by at least 5 reads on the two mtDNA strands (H and L) per biological replicate (*N* = 15 samples per protocol), in samples processed with (top) fragmentation protocol and (bottom) BamHI-based protocol. (**C**) Distribution of the average read depth per mtDNA position in samples processed with (top) fragmentation protocol and (bottom) BamHI-based protocol (*N* = 15 samples per protocol). Samples in (A−C) are the same processed in parallel with either protocol

Under the conditions outlined above, the fragmentation-based method showed a mapping bias on the L-strand (L-strand_FRAG_ = 46.12% ± 5.13, H-strand_FRAG_ = 53.87% ± 5.13, percentage of aligned reads mean ± sd; Anova one-way test *P* ≤ 0.001, Figure 2A, Supplementary Figure S6A) with six samples having <100% coverage (Figure 2B). On the contrary, the BamHI-based protocol did not show significant mapping or coverage bias (L-strand_BAMHI_ = 50.67% ± 4.07, H-strand_BAMHI_ = 49.32% ± 4.07, percentage of aligned reads mean ± sd; *P* = 0.36, Figure 2A, B, Supplementary Figure S6A). The average mtDNA read depth was higher in the samples processed with the BamHI-based protocol (Frag. = 23.83*x* ± 4.33, BamHI = 131.73*x* ± 8.15, mean ± sd; *P* ≤ 0.0001, Figure 2C, Supplementary Figure S6B), with almost half of the mitochondrial reads mapped as full-length molecules (≥15 000 bp; 42% ± 12 of BamHI reads versus 2% ± 2 of Frag. reads, Supplementary Figure S3D). Overall, these results suggest that our custom-made BamHI ONS protocol is more efficient in achieving full-length mtDNA enrichment and higher mtDNA read depths than the standard Nanopore library preparation.

### MtDNA sequencing and the detection of heteroplasmic variants with ONS

Next, we set out to validate the BamHI-ONS protocol for mtDNA sequencing including heteroplasmy detection. We used high-depth Illumina MiSeq sequencing of mtDNA to determine the major alleles and accurately measure heteroplasmy levels (mean read depth = 2769*x*, min = 318*x*, max = 5559*x*; Supplementary Table S6) in the primary and cancer cell lines (Table 1, Supplementary Results). Variant calling with ONS detected 99.5% (*N* = 739/743) of the homoplasmic variants (het. ≥ 95%) also detected by the Illumina sequencing, enabling reliable haplogroup predictions (Supplementary Results, Table S6 and Figure S7). As reported previously for exome sequencing (32), higher ONS read-depths were required to reliably measure heteroplasmy levels detected by high-depth Illumina sequencing (Supplementary Figure S8).

### Establishing the methylation detection strategy with ONS

We first assessed the accuracy of the methylation calling on mtDNA by sequencing a near complete PCR amplicon of human mtDNA (negative control, NC, 0% methylated) and a corresponding positive control generated *in vitro* with a recombinant CpG methyltransferase (PC, 100% methylated; Supplementary Figure S1). We used Nanopolish software (25) to call methylation on PC and NC, which generated log-likelihood ratio (LLR) values of CpG methylation (Supplementary Figure S9A). A site is considered methylated when its LLR is above a certain threshold. To choose the most accurate methylation calling cut-off for mtDNA, we: (i) determined the ratios of true and false positives by varying LLR thresholds values (following previous procedures (25), Materials and Methods) and calculating a receiving operating characteristic (ROC) and (ii) methylation calling accuracy (intended as proportion of true calls; Supplementary Figure S9B-C). The ability to distinguish between mtDNA unmethylated and methylated sites was measured by the area under the ROC curve (AUC), which was equal to 0.97 (Supplementary Figure S9B). With the default Nanopolish LLR threshold (≥2.5), an accuracy of 97.7% could be achieved (Supplementary Figure S9C). Hence, we chose a more stringent methylation calling threshold (LLR ≥ 5) yielding an accuracy of 99% (Supplementary Figure S9C). Also, by looking at methylation profiles of NC, we identified 13 CpG positions that showed methylation levels above 2 standard deviations from the NC mean (likely false positives, Supplementary Table S3 and Figure S9D), which were investigated further.

We then performed CpG methylation calling followed by differential methylation (DM) analysis (18), in all the cell lines and primary fibroblasts (Table 1, Materials and Methods). First, we checked the methylation levels of the 13 likely false positives we had identified in the NCs, and we found them to be methylated consistently in all our samples analysed (Supplementary Table S3). Hence, we removed the 13 positions from all subsequent ONS sequencing experiments results.

**Figure 3.**
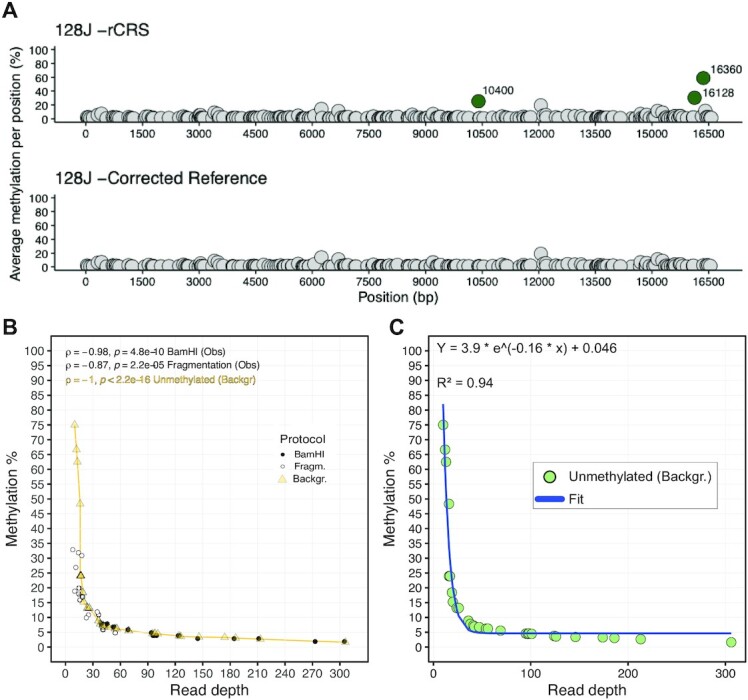
Methylation detection strategy with ONS. (**A**) Example of methylation calling artefacts introduced when using hg38 as reference (which includes the mitochondrial reference sequence rCRS) (top) instead of a sample-specific consensus sequence (bottom). In green are highlighted the sample-specific differentially methylated positions which disappear upon reference correction. (**B**) Correlation between average read depth and average methylation percentage in samples processed with fragmentation- and BamHI-based protocols (Obs) and in unmethylated datasets simulated from the negative control (Backgr). Circles represent a sample sequenced with either sequencing protocol (*N* = 15 per protocol). Triangles represent an unmethylated dataset simulated from the negative control. Spearman's rank correlation coefficient and two-sided *P*-values are shown. (**C**) Green circles represent correlation between average read depth and average methylation percentage in 30 unmethylated datasets simulated from negative controls. The blue solid line represents the fitted line (exponential decay function) that describes such distribution, corresponding to the methylation background noise (Backgr). The formula describing the fit and *R*^2^ correlation calculated with the goodness of fit test are shown.

In the cell lines, the analysis revealed several other possible differentially methylated CpGs (DM-CpGs; Supplementary Table S7). However, close scrutiny revealed that an haplogroup-defining mtDNA variant always fell within a ± 5 bp window from a possible DM-CpG, prompting us to hypothesize that the haplogroup variants influenced Nanopolish methylation calling (Materials and Methods). To test this, we generated a new reference for methylation calling based on a mtDNA consensus sequence of the major mtDNA alleles identified with Illumina MiSeq sequencing. DM analysis using sample-specific consensus sequences entirely removed the apparent methylation signal detected earlier (Figure 3A, Supplementary Table S7). Therefore, we set out to perform methylation calling always using a sample-specific consensus sequence. We also compared the level of DM on mtDNA molecules carrying the m.3243A>G mutation to wild-type molecules in the same two primary fibroblasts lines, but saw no difference in CpGs methylation between the two molecular species (Supplementary Table S7).

We observed a negative correlation between read depth and methylation signal in the samples used to test the enrichment protocol (Table 1), which suggested that the observed methylation was background noise intrinsic to the technology (Figure 3B, Supplementary Results). To test this hypothesis, we performed subsampling from the NC and generated simulated unmethylated ONS datasets (‘Background’). These matched the read depths obtained with both fragmentation and BamHI-based protocol-derived experiments (‘Observed’) (Figure 3C). We then inferred a model that best fitted the simulated data (*R*^2^ = 0.94, Figure 3B, Materials and Methods) which we used to estimate the background noise in methylation calling of all the ONS experiments we have performed in this study.

### ONS-based CpG methylation analysis of mtDNA in human cell lines and tissues

**Figure 4.**
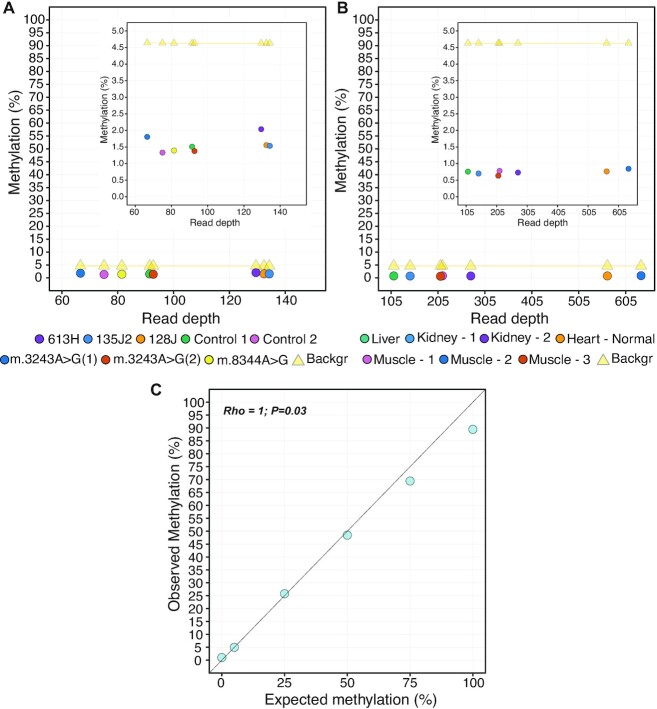
ONS analysis on human cell lines and tissues reveals absence of relevant CpG methylation levels on mtDNA. (A, B) Scatterplots showing the relationship between average read depth and average methylation percentage in samples processed with BamHI protocol. Circles in (**A**) represent an average of all mtDNA positions in either five (cell lines) or three (primary fibroblasts) biological replicates. Circles in (**B**) represent an average of all the mtDNA positions in human tissues from seven different individuals. Yellow triangles in (A) and (B) represent the background noise. Inset plots show magnification of the data shown. (**C**) Correlation between the expected and observed methylation levels calculated on methylated controls generated by mixing PC and NC. Spearman's test, *P*-value and rho are shown.

Using a sample-specific mtDNA reference sequence for methylation calling we looked for evidence of mtDNA methylation in all three cancer cell lines (*N* = 5 biological replicates) and three primary fibroblast lines (*N* = 3 biological replicates; Figure 4A, Supplementary Table S7; Methylation_C_LINES/FIB_ = 1.3−2%; min−max). In each case, the apparent methylation values were below the estimated background noise level.

To extend these findings to *in vivo* samples, we sequenced seven fresh human tissues of different healthy individuals (Table 1, Supplementary Table S5). Again, we observed that the apparent methylation levels were below the estimated background noise, even at higher read depths compared to the cell lines and primary fibroblasts (Figure 4B, Supplementary Table S7; Methylation_TISSUES_ = 0.6–0.8%; min-max).

Finally, we sought for conclusive evidence that ONS is capable of identifying methylation above the background level. To do that, we generated and sequenced with ONS 4 additional control samples with expected methylation levels of 5%, 25%, 50% and 75%, by mixing the PC and NC (Materials and Methods). Results of this analysis revealed that the expected methylation levels were correctly measured with ONS (Rho = 1, *P* = 0.003, Spearman's rank test, Figure 4C).

## DISCUSSION

The discovery of mitochondrially-targeted methyltransferases (33–35) implied that mtDNA could be methylated. In addition to a role in modulating mitochondrial gene expression, multiple studies report mtDNA CpG methylation as a biomarker of ageing (36), environmental exposure to tobacco smoke (37), cancer (38) and neurological diseases (39,40). Currently, quantitative analysis of CpGs is mostly based either on mass spectrometry or on the bisulfite treatment of gDNA (bisulfite pyrosequencing and WGBS). While the first method is the most sensitive in determining the general CpG methylation level of a given sample, it lacks information about the position of individual methylated residues (41). On the other hand, while bisulfite-based technologies resolve the CpG methylation at a single-base level, they are susceptible to the introduction of biases due to the selective degradation of cytosine-rich sequences (both nuclear and on the L-strand of mtDNA) (13,42). Despite this, multiple groups have continued to investigate mtDNA methylation using bisulfite-based technologies, without accounting for presence of alignment biases (35,43).

In an attempt to resolve this controversy, we studied 55 publicly available WGBS datasets part of the Roadmap Epigenome Project (19), focussing on describing per-strand sequencing metrics and how these affect the methylation profile of mtDNA. Our analysis confirmed a marked per-strand bias with an impact on global mtDNA CpG methylation levels quantification.

To overcome WGBS limitations, we developed an accurate and reproducible protocol to investigate mtDNA methylation using ONS, and tested our method against the standard ONS library preparation protocol based on random fragmentation. Our protocol is based on selective restriction digestion by BamHI followed by selection of longer sequences, which results in an enrichment for native full-length mtDNA thereby minimising the potential for NuMTs contamination. Comparing our results with Illumina sequencing, we found that our protocol allows the correct calling of the vast majority of homoplasmic and high-heteroplasmy (>95%) mtDNA alleles, including pathogenic mutations.

Our analysis also revealed that the methylation calling with Nanopolish is influenced by the presence of mtDNA variants surrounding the CpG residue. In light of this, we recommend a careful review of previously identified methylated positions and of differential methylation results (44) in the context of the nucleotide reference sequence. This is also likely to be an issue for nuclear DNA, although we have not formally studied this here. It is not clear why the 13 positions listed in Supplementary Table S3 appeared to be methylated in every one of the samples we analysed. However, local sequence context is known to influence methylation calling, as we have shown for mtDNA Figure 3, and has been attributed to systematic errors introduced by the Nanopolish software (45).

Finally, our study indicates that, after removing the technical biases and regressing out the background noise, no residual CpG mtDNA methylation could be identified with ONS across multiple human tissues and cell lines. These findings add to emerging evidence (3–5,11) that CpG methylation is not occurring on human mtDNA. Although our findings do not exclude the possibility of very low levels of mtDNA methylation, these are unlikely to be biologically relevant. The exact nature of this background noise is an interesting matter of debate. The residual signal could come from methylated NuMTs sequences which aligned to the mtDNA reference despite our filtering applied before and after alignment. Against that, we saw the noise despite minimising the potential for NuMTs contamination by developing a protocol focussed on full-length mtDNA sequencing. It is also unlikely that spontaneous background methylation *in vivo* explains the residual signal, because the concentrations of SAM necessary to introduce mutations are ∼16-fold greater than the levels measured in mitochondria (46,47). It therefore seems more likely that the noise arises from random variations in the ONS electric signal at the moment it is generated and registered. Such small variations would possibly be interpreted by Nanopolish as background methylation.

Importantly, the protocol we have developed has potential applications beyond CpG methylation, including mtDNA variant calling and measuring other types of epigenetic modifications (e.g. m6A methylation) at the single nucleotide level (48,49).

## DATA AVAILABILITY

The raw DNA sequencing data in this manuscript has been deposited on the SRA archive and is accessible using the BioProject accession number PRJNA763486. The MToolBox pipeline used for mitochondrial variant calling and code for filtering VCF files are available as GitHub branch of the MToolBox repository: https://github.com/mitoNGS/MToolBox/tree/MToolBox_Nanopore. Code used for plotting and data analysis is available at https://github.com/ib361/scripts_paper.

## Supplementary Material

gkab1179_Supplemental_Files
