# Supplementary material for: Single-molecule mitochondrial DNA sequencing shows no evidence of CpG methylation in human cells and tissues
Source: Nucleic Acids Res. 2021 Nov 29;49(22):12757–68. doi: 10.1093/nar/gkab1179 (PMC8682748; doi:10.1093/nar/gkab1179)
Supplement: gkab1179_Supplemental_Files [file gkab1179_supplemental_files.zip › Supporting_information_revised.pdf]

Supporting Information

Single-molecule mitochondrial DNA sequencing reveals absence of CpG methylation in human cells and tissues

Supplementary Results

Variant calling results

In addition to the cybrids cell lines, we sequenced (using the BamHI protocol) mtDNA from primary fibroblasts including patients carrying known heteroplasmic pathogenic mutations and healthy control subjects (without known mtDNA mutations; “Control 1”, “Control 2”; **Table 1**, **Supplementary Table S5**). Specifically, we sequenced one patient carrying the heteroplasmic m.8344A>G/*MT-TK* mutation (“m.8344A>G"), causative of myoclonic epilepsy with ragged red fibers (MERRF) syndrome. We also sequenced 2 patients carrying the m.3243A>G/*MT-TL1* mutation (“m.3243A>G”), known to cause Mitochondrial Myopathy, Encephalopathy, Lactic Acidosis, Stroke-Like Episodes (MELAS[37,38], N = 3 technical replicates each, **Table 1**, **Supplementary Table S5**).

Variant calling confirmed ONS homoplasmic variants, haplogroup predictions and the detection of the known single nucleotide heteroplasmic variants in the majority of biological replicates of primary fibroblasts (N=2/3 for m.3243A>G (1), N=3/3 m.3243A>G (2), N=2/3 m.8344A>G; **Supplementary Table S6** and **Figure S7**). Since we observed a base calling accuracy of ~90% in our samples sequenced with ONS (**Supplementary Figure S4**), we set a stringent threshold of 10% heteroplasmy to call for mtDNA variants. On average, we found 60 mtDNA variants with ≥ 10% heteroplasmy per cell line with ONS, of which 28 (~47%) were confirmed with Illumina Miseq (**Supplementary Figure S7** right plot). These were mostly highly heteroplasmic or homoplasmic variants ($heteroplasmy_{ONS}$ = 93% ± 17%; $heteroplasmy_{Miseq}$ = 96% ± 15%; mean ± sd; **Supplementary Figure S7** left plot). The remaining unconfirmed mtDNA variants were low heteroplasmic ($heteroplasmy_{ONS}$ = 16% ± 11%, mean ± sd; **Supplementary Figure S7**).

Supplementary Figures

Figure S1. Experimental setup of mitochondrial negative and positive controls and ONS methylation analysis

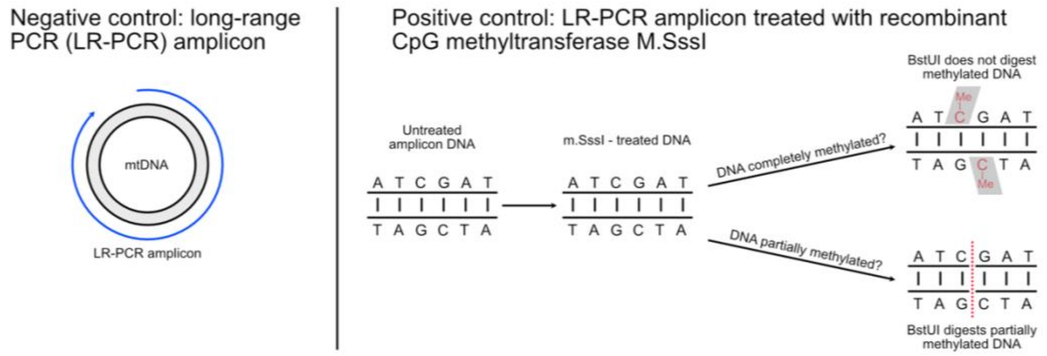

Figure S2. WGBS alignment bias and methylation analysis

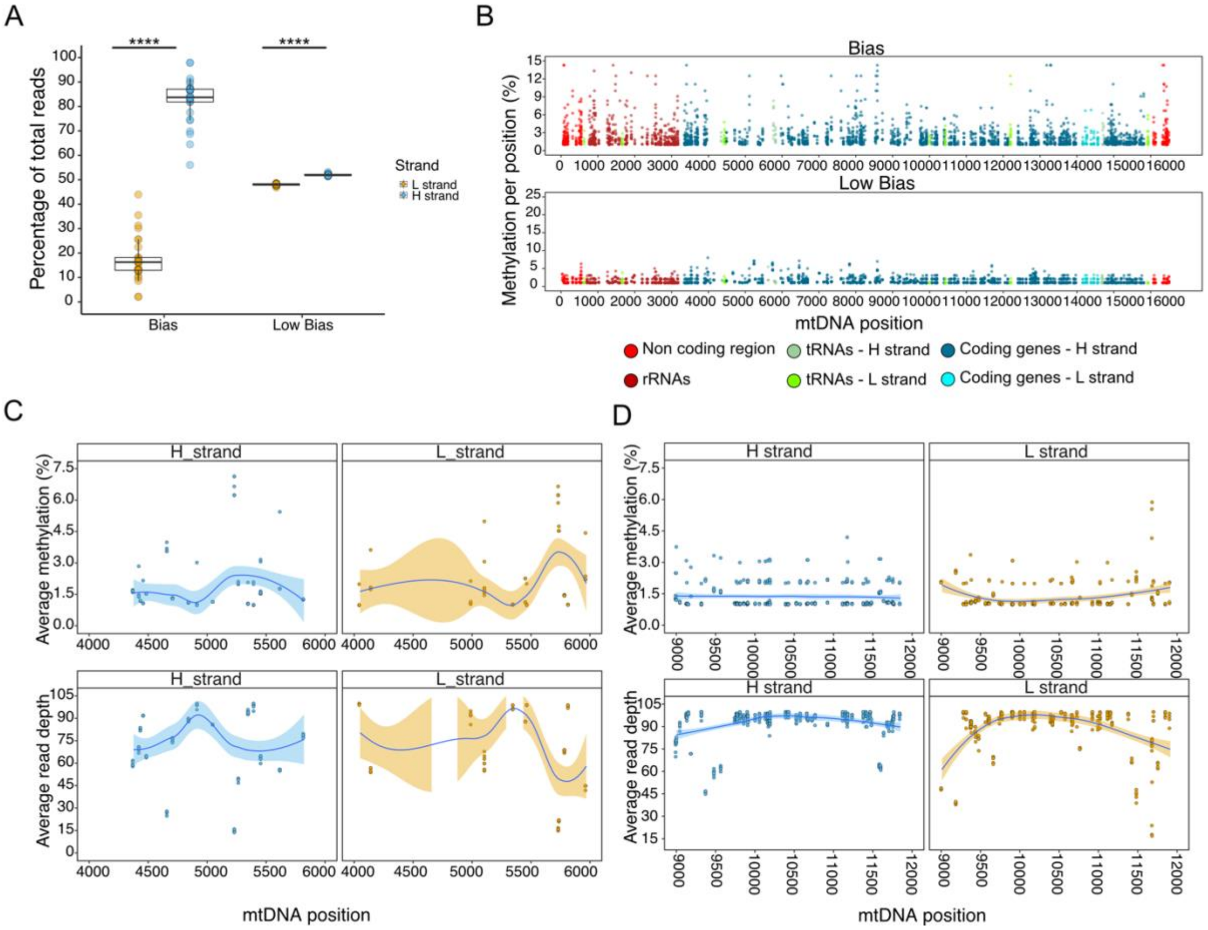

a) Percentage of reads aligned to mtDNA, divided by bias group. Boxplot shows the percentage of reads aligned to the mtDNA reference. The lower and upper hinges correspond to the first and third quartile of the distribution, with median in the centre and whiskers span no further than 1.5*interquartile range. Stars indicate significance (****: two-sided $P \leq 0.0001$, Wilcoxon test). **b**) Distribution of the methylation percentage per mtDNA position in CpG context, in (top) Bias (N = 32) and (bottom) Low Bias (N = 23) groups. Each dot represents every CpG in every sample. Methylation values are expressed in % of methylation. **c-d**) CpG methylation and read depth profiles of a 2kb (**c**) and 3kb (**d**) mtDNA genome region, per each position in the Low Bias sample group, divided by mtDNA strand (H and L). Each dot represents all the CpGs in the specific area in all the Low Bias samples. Methylation values are expressed in % of methylation. Blue lines indicate the mean over all the data points (calculated using the “loess” *geom_smooth* R function) and shaded surrounding regions represent 95% confidence interval.

Figure S3. Design and assessment of an ONS-based protocol for mtDNA enrichment and analysis

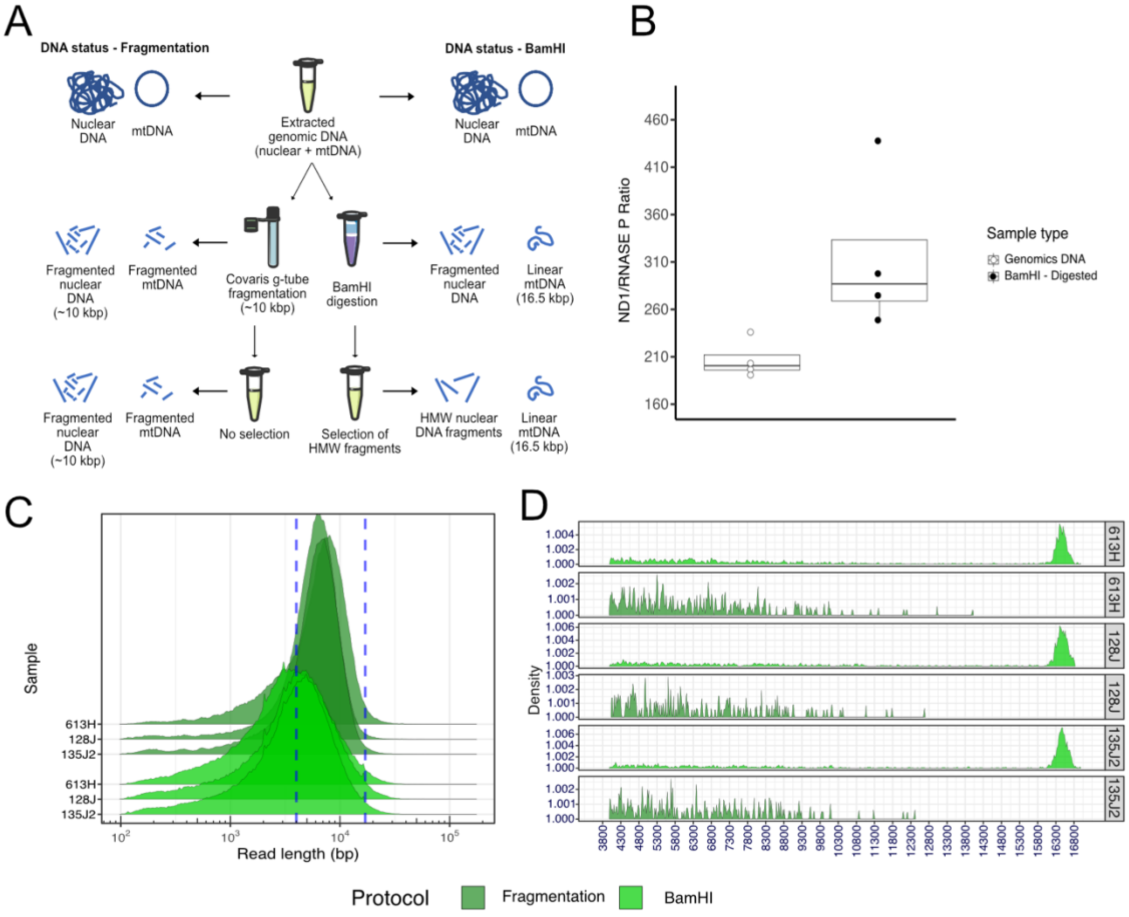

a) Overview of the workflow used to process samples using (left) standard ONT fragmentation protocol and (right) BamHI-based protocol. **b**) Ratio of signal from the mitochondrial MT-ND1 over RNASE P ddPCR probes in undigested genomic DNA and BamHI-digested genomic DNA. N = 4 for each protocol used. Star indicates significance (*: two-sided $P \leq 0.05$, Wilcoxon test). **c**) Distributions of the total sequenced reads in 3 cybrids cell lines prepared with either fragmentation or BamHI-based protocols. Blue dashed lines correspond to the chosen cut-off for read filtering at 4000bp and 17000bp. **d**) Distribution of the mtDNA aligned reads filtered by length (4000bp -17000 bp) in 3 cybrids cell lines prepared with either fragmentation or BamHI-based protocols.

Figure S4. Nanopore sequencing metrics

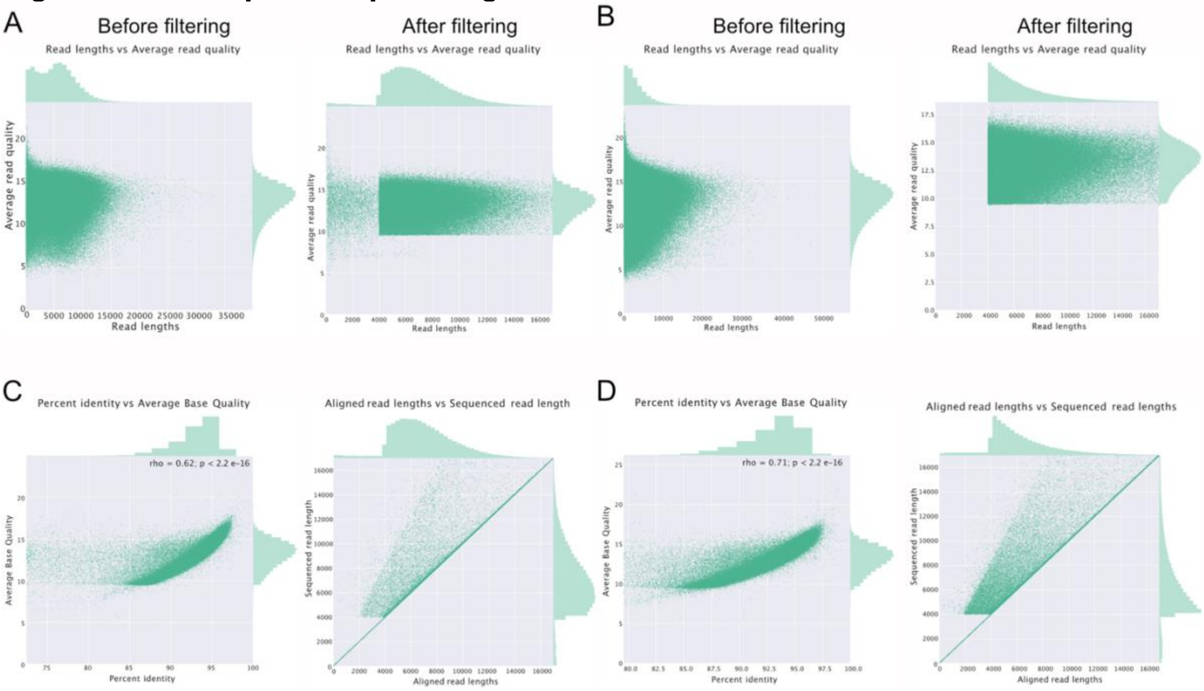

a) Plots show the correlation between read lengths and read quality scores in one sample processed with the fragmentation protocol before filtering (left) and after filtering (right). **b**) Plots show the correlation between read lengths and read quality scores in one sample processed with the BamHI-based protocol before filtering (left) and after filtering (right). **c**) Plots show the correlation in one sample processed with the fragmentation protocol between percent identity to the reference sequence and average quality of the reads (left), and correlation between aligned read lengths and sequenced read lengths (right). **d**) Plots show the correlation in one sample processed with the BamHI protocol between percent identity to the reference sequence and average quality of the reads (left), and correlation between aligned read lengths and sequenced read lengths (right).

Figure S5. ONS alignment metrics: percentage of reads aligned on mtDNA

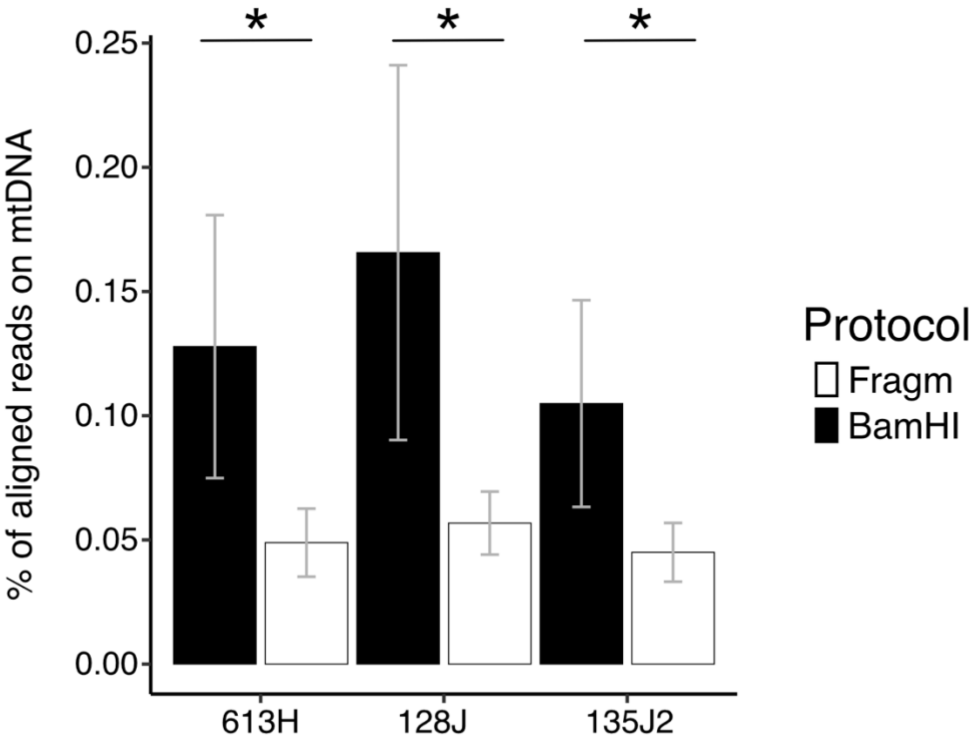

Percentage of aligned reads on mtDNA and in fragmentation and BamHI sequenced samples (N=5 each). Stars indicate significance (*: two-sided P ≤ 0.05, Student's t-test).

Figure S6. ONS alignment metrics: bias quantification and read depths

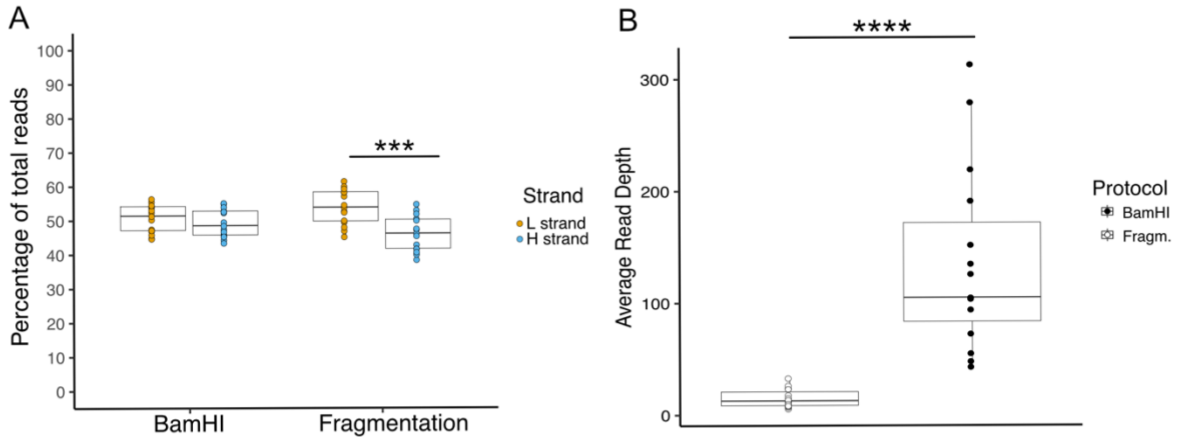

a) Percentage of reads aligned to mtDNA, divided by strand and library preparation protocol (N = 15 per protocol). Stars indicate significance (***: P ≤ 0.001, Anova one-way test). **b**) Average read depth per sample observed in the same sample pool processed using either fragmentation protocol (left) or BamHI-based protocol (right). N = 15 per protocol. Stars indicate significance (****: two-sided *P* = ≤ *0.0001*, Wilcoxon test).

Figure S7. ONS-based variant calling of mtDNA

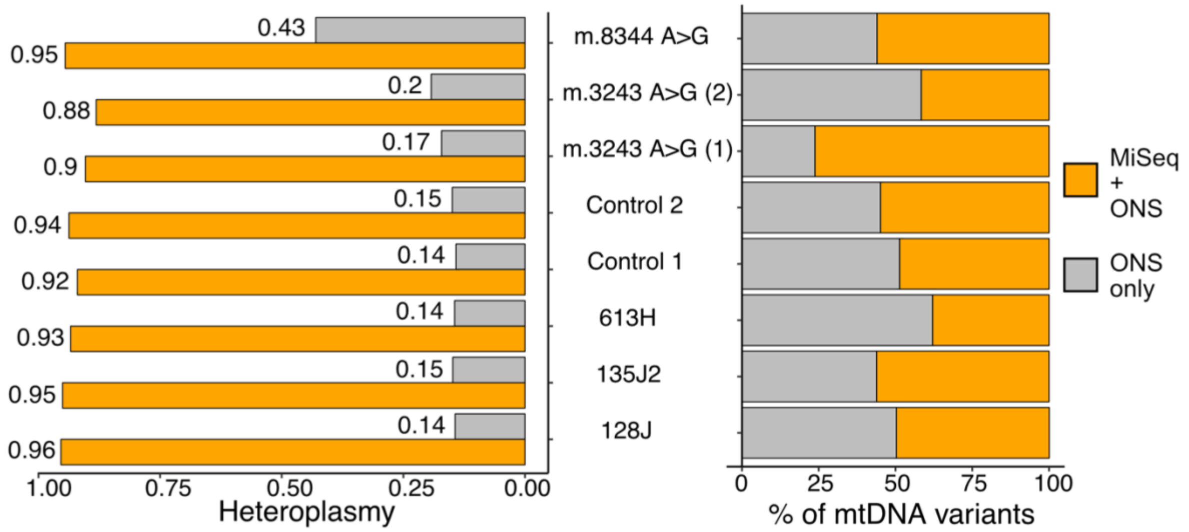

Variant calling statistics per cell line analysed. Heteroplasmy (left) and percentage of single nucleotide mtDNA variants (right) identified with either Illumina Miseq and ONS or ONS only. Values are means calculated across all biological replicates per cell line analysed (N = 5 for 613H/128J/135J2 and N = 3 for Control1/Control2/m.3243A>G (1)/m.3243A>G (2)/m.8344A>G).

Figure S8. Differences in heteroplasmy detection as a function of the ONS read depth

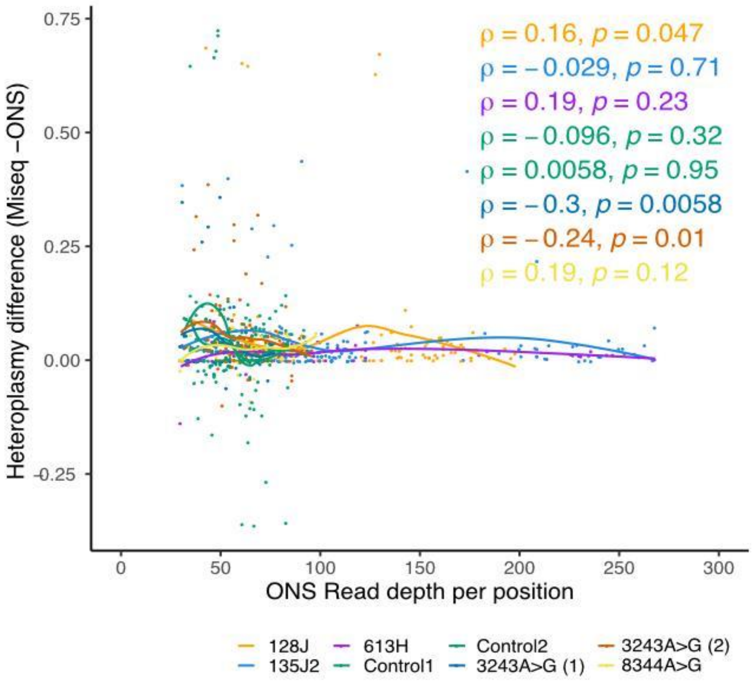

Scatterplot showing a correlation between differences in heteroplasmy values quantified with Illumina Miseq and ONS (calculated as Miseq heteroplasmy - ONS heteroplasmy), for each single nucleotide mtDNA variant detected with both techniques, and ONS read depth per position. Colours correspond to the different samples analysed (N = 5 for 613H/128J/135J2 and N = 3 for Control1/Control2/m.3243A>G (1)/m.3243A>G (2)/m.8344A>G), with lines indicating mean over all the data points in each sample (calculated using the “loess” *geom_smooth* R function). Spearman's rank two-sided p-values and rho coefficients are shown.

Figure S9. Assessing the sensitivity of ONS-based methylation calling strategy

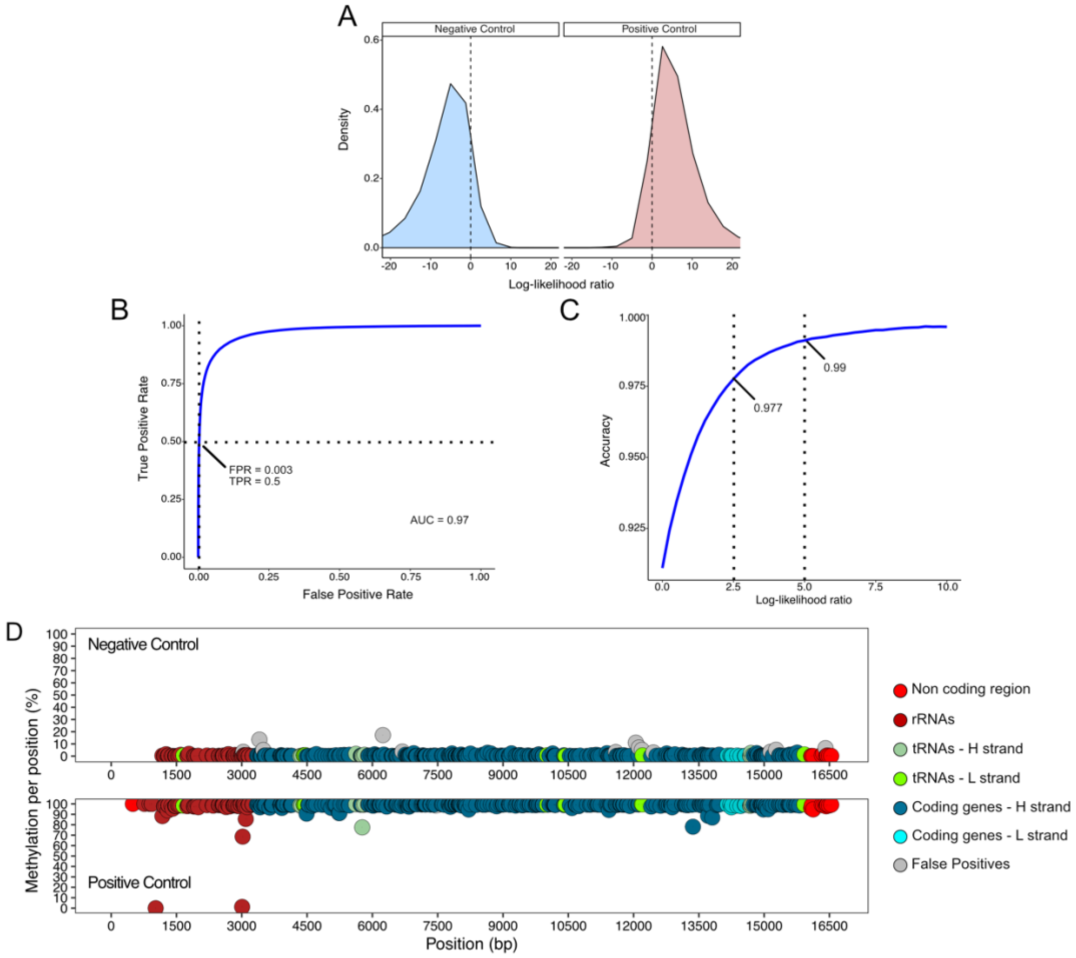

a) Log-likelihood ratio values of methylation calculated by Nanopolish, using the positive and negative controls. The log-likelihood ranges between -20 and 20 (used to build the Receiver operating characteristic (ROC) curve) are shown. **b**) ROC curve calculated by changing the methylation call log-likelihood ratio threshold from a value of -20 to 20, with a step of 0.25. The dash lines are drawn at FPR (False Positive Rate) and TPR (True Positive Rate) values obtained by setting the ratio equal to 5. AUC = area under the curve. **c**) Methylation call accuracy calculated at increasing values of log-likelihood ratio (ranging between 0 and 10). The dash lines indicate the accuracy achieved at the ratio equal to 2.5 (accuracy = 0.977) and 5 (accuracy = 0.99). **d**) Distribution of the methylation percentage per mtDNA position in CpG context, in (top) negative and (bottom) positive controls. Methylation values are expressed in % of methylation. Grey values represents the 13 positions identified as false positives.

Supplementary Tables

Supplementary Table S1. Primers and probes list

**Primers**			
Primer name	Forward 5' - 3'	Reverse 5' - 3'	Used for
2F	TGTAAAACGACGGCCAGTTTAAAACTCAAAGGACCTGGC	-	LR-PCR
D1R	-	CAGGAAACAGCTATGACCAGGGTGATAGACCTGTGATC	LR-PCR
MT-ND1	GGGTTCATAGTAGAAGAGCGATGG	ACGCCATAAAACTCTTCACCAAAG	dPCR
RNASE P	AGATTTGGACCTGCGAGCG	GAGCGGCTGTCTCCACAAGT	dPCR
Illumina_primer_1_Fw	CATCCGTATTACTCGCATCAG	-	
Illumina_primer_1_Rev	-	TTGGCTCTCCTTGCAAAGTT	
Illumina_primer_2_Fw	TATCCGCCATCCCATACATT	-	
Illumina_primer_2_Rev	-	AATGTTGAGCCGTAGATGCC	
**Probes**			
Probe name	Fluorophore	Sequence 5' - 3'	Quencher
MT-ND1	HEX	ACCCGCCACATCTACCATCACCCTC	BHQ_1
RNASE P	FAM	TTCTGACCTGAAGGCTCTGCGCG	BHQ_1

Supplementary Table S2. Generation of positive controls at intermediate methylation levels

**Expected Methylation**	**Negative Control (ng)**	**Positive Control (ng)**	**Total**
0%	1 µg	0	1 µg
5%	50	950	1 µg
25%	750	250	1 µg
50%	500	500	1 µg
75%	250	750	1 µg
100%	0	1 µg	1 µg

Supplementary Table S3. False positive positions and methylation values.

**MtDNA position**	**Methylation Frequency In NC**	**Methylation Frequency in Cell Lines and Tissues (average)**	**Standard deviation in Cell Lines and**

			Tissues
3034	0.035	0.05	0.07
3405	0.135	0.09	0.07
3494	0.05	0.11	0.11
6241	0.172	0.07	0.04
6688	0.037	0.13	0.06
11590	0.033	0.06	0.04
12052	0.11	0.25	0.13
12123	0.07	0.08	0.08
12190	0.051	0.03	0.03
12455	0.03	0.08	0.06
15146	0.035	0.05	0.07
15274	0.052	0.05	0.04
16410	0.065	0.06	0.06

These positions were first identified in the NCs as being +2 sd higher than the NCs average methylation levels.

Supplemental Data Legends

Supplementary Table S4: List and metrics of WGBS samples that passed quality control

The table includes for each bias group (tabs “Bias” and “Low Bias”): sample IDs and descriptions, total sequenced throughput (in basepairs), average mitochondrial read depths measured with Samtools depth, number and percentage of reads aligned to each mitochondrial strand, coverage per strand.

Supplementary Table S5. List and metrics of samples sequenced with ONS in this study.

The table includes: sample IDs and descriptions, library preparation method used for sequencing with ONS (fragmentation/BamHI), total sequenced throughput (in basepairs), average mitochondrial read depths measured with Samtools depth, number and percentage of reads aligned to each mitochondrial strand, coverage per strand.

Supplementary Table S6. Illumina Miseq and ONS sequencing metrics and variant calling

The table includes: Miseq and ONS sequencing read depth and coverage (percentage of mtDNA covered by at least one read) calculated by running the MToolBox pipeline (tab “Read depth, coverage, haplo predictions”); haplogroup predictions calculated with MToolBox and

Haplogrep2 (tab “Read depth, coverage, haplo predictions”); list of mtDNA SNVs identified with ONS, with read depth ≥ 30 and heteroplasmy ≥ 10% and corresponding *per*-base read depth and heteroplasmy quantified with Illumina Miseq (tab “mtDNA variants”). Pathological mutations (m.3243A>G, m.8344A>G) identified by ONS are indicated in red; list of homoplasmic mtDNA SNVs (het. ≥ 95%) identified with Illumina sequencing and confirmed with ONS (tab “Homoplasmies Illumina vs ONS”).

Supplementary Table S7: ONS methylation analysis results

The table includes: average methylation values per position calculated on cell lines, primary fibroblasts and tissues using the consensus sequences (tabs “Cell lines meth. values" and “Primary fib. meth. values”, “Tissues meth. values”); differential methylation analysis results performed on average methylation values calculated either with rCRS (tab “Diff. meth. on rCRS) or the consensus sequences (tab “Diff. meth. on consensus); differential methylation analysis results performed on average methylation values of mitochondrial CpGs found on either WT or mutated mitochondrial DNA sequenced in the 2 m.3243A>G primary cell lines (“m.3243 A>G (1) WT Vs Mut SS res” “m.3243 A>G (2) WT Vs Mut SS res”).
